# Comparison of tissue damage and inflammation for robotic laparoscopy and conventional laparoscopy in early endometrial cancer

**DOI:** 10.3389/fmed.2024.1492469

**Published:** 2024-11-01

**Authors:** Shengnan Meng, Yanling Cao, Qingwei Shen, Ling Dong, Nan Wang

**Affiliations:** ^1^Department of Gynecology, Hospital of Traditional Chinese Medicine of Qiqihar, Qiqihar, China; ^2^Department of Gynecology, Shanghai First Maternity and Infant Hospital, Shanghai, China; ^3^Department of Obstetrics and Gynecology, Luodian Hospital in Baoshan District, Shanghai, China

**Keywords:** tissue damage, inflammation, robotic laparoscopy, conventional laparoscopy, early endometrial cancer

## Abstract

**Introduction:**

This study was to analyze the dynamics of tissue damage and inflammatory response markers perioperatively and whether these differ between robotic laparoscopy and conventional laparoscopy in early endometrial cancer.

**Methods:**

In a randomized controlled trial conducted at SHANGHAI FIRST MATERNITY and INFANT HOSPITAL, eighty women with early-stage, low-risk endometrial cancer were randomly assigned to receive either robotic or conventional laparoscopy. Blood samples were collected at admission, immediately before surgery, 2 h after surgery, 24 h after surgery, 48 h after surgery, and 1 week after surgery. The samples were analyzed for various biomarkers associated with inflammatory processes and tissue damage. These included high-sensitivity C-reactive protein (hs-CRP), white blood cell count (WBC), platelet count, interleukin-6 (IL-6), cortisol, creatine kinase (CK), and tumor necrosis factor-alpha (TNF-*α*). These markers provide insights into the underlying physiological responses and potential tissue-level changes within the study participants.

**Results:**

There was no significant difference in clinical and preoperative data between two groups. The results showed that the patients who underwent robotic laparoscopy had a longer pre-surgical time compared to the conventional laparoscopy group. However, the robotic group had shorter operating times, quicker vaginal cuff closures, and lower estimated blood loss compared to the conventional laparoscopy group. The hospital stays, Visual Analog Scale (VAS) score and drainage volume on the first day after operation were lower in robotic group compared to conventional laparoscopy group. hs-CRP, WBC, IL-6 and cortisol were significantly lower in the robotic group, though the differences were transient.

**Discussion:**

This study demonstrated that robotic laparoscopy, used in early endometrial cancer treatment, leads to a reduced inflammatory response, less tissue damage, and lower stress levels, as evidenced by decreased levels of hs-CRP, IL-6, and cortisol, compared to conventional laparoscopy. These findings suggest that robot- laparoscopy may facilitate a quicker recovery and improve patient-reported outcomes.

## Introduction

Postoperative tissue damage and inflammatory responses are critical determinants of recovery, involving complex cascades of biochemical processes. Evaluating these responses involves monitoring fluctuations in blood levels of inflammatory proteins, immune cells, stress hormones, and markers of tissue damage. Research has shown that minimally invasive methods for hysterectomy, such as vaginal or laparoscopic approaches, lead to reduced tissue damage and attenuated inflammatory responses when compared to the traditional abdominal hysterectomy (1–3). Furthermore, research indicates that the type of hysterectomy and anesthesia used can significantly influence the immune response. Laparoscopic hysterectomy is associated with a less pronounced inflammatory response and a relatively minor impact on cellular immunity when compared to traditional abdominal hysterectomy (4, 5).

Since receiving approval from the U.S. Food and Drug Administration for gynecological procedures in 2005, robot-assisted laparoscopy, particularly through the da Vinci Robotic Surgery System, has seen increased adoption in gynecologic surgeries. Conventional laparoscopy often struggles with limited visualization and reduced dexterity. In contrast, the da Vinci surgical robot offers high-definition 3D imaging and precise instrument control, addressing the shortcomings of conventional laparoscopy (6). Moreover, given prior research that compares inflammatory markers following laparoscopic versus open hysterectomy, it might be anticipated that robotic laparoscopy would exhibit fewer adverse pathophysiological effects (7). Nonetheless, there is a scarcity of published randomized controlled trials concerning robotic laparoscopy within the field of gynecologic oncology.

The aim of this study was to analyze the dynamics of tissue damage and inflammatory response markers perioperatively and whether these differ between robot-assisted laparoscopy and conventional laparoscopy in early endometrial cancer.

## Materials and methods

### Study population

A prospective, randomized controlled study was conducted at the Department of Gynecology, Shanghai First Maternity and Infant Hospital, Shanghai, China. The study enrolled women with early-stage endometrial cancer (FIGO stage I, low-risk endometrioid adenocarcinoma, FIGO IA and IB) who were scheduled to undergo hysterectomy, bilateral adnexectomy, and pelvic and para-aortic lymph node dissection between September 2022 and October 2023. After obtaining informed consent, the participants were randomly assigned to either robotic-assisted laparoscopy (*n* = 40) or conventional laparoscopy (*n* = 40) groups.

All participants underwent standard preoperative evaluation, including routine pre-admission testing, and received identical information regarding their care and perioperative guidance. Anesthesia, postoperative analgesia, and perioperative fluid therapy were also standardized and similar across both groups. The robotic-assisted laparoscopic procedures were performed using the da Vinci® Surgical System, with four robotic ports and three robotic arms. All operations were carried out by board-certified gynecological oncology surgeons. Following hospital discharge, a research nurse maintained regular contact with the patients to collect blood samples and monitor for any potential complications.

### Inflammatory and tissue damage markers

We selected a panel of markers that has previously been shown to reflect acute inflammation and response to tissue damage after surgery and stress (8–10). The panel consisted of white blood cells (WBC), platelets, high sensitivity C-reactive protein (hs-CRP), interleukin-6 (IL-6), creatine kinase (CK), tumor necrosis factor (TNF)-*α* and cortisol.

### Blood samples-time frame

Markers of tissue damage and inflammatory response were assessed using peripheral venous blood samples, which were collected at multiple time points: at admission (Time 1), on the surgery day before the procedure (Time 2), and post-surgery at 2 hours (Time 3), 24 h (Time 4), 48 h (Time 5), and 1 week (Time 6). These samples were centrifuged within an hour of collection and the aliquots were then stored at −70°C. All analyses were conducted in a single session, except for cell counts, which were done immediately after collection. The laboratories conducting these analyses were not aware of the surgical method used.

### Analysis of laboratory data

The WBC and platelets were analyzed by a CellDyn Sapphire Hematology Analyzer (Abbott Laboratories, IL, United States). hs-CRP were measured using a commercially available immunonephelometric kinetic assay (BN ProSpec; Siemens, Tarrytown, NY, United States) using Cardiophase hs-CRP reagents. The cortisol levels were measured using a Cobas e 602 analyzer as part of a Cobas 8,000 modular analysis series using latex particle-enhanced immunoturbidimetric assay (Roche Diagnostics, Germany). CK was measured using a Cobas e 701 analyzer as part of Cobas 8,000 modular analysis series (Roche Diagnostics, Germany) using creatine kinase reagents from Roche. IL-6 was were assessed by enzyme-linked immunosorbent assay using cytokine-specific kits (Abcam, Cambridge, United Kingdom).

### Statistical analysis

Quantitative data are expressed as the mean value ± standard deviation, while qualitative data are expressed as frequency (percentage). The independent two-sample *t*-test was used for comparisons between cohorts. The chi-square test or Fisher’s exact test was used to compare categorical variables, as applicable. Two-sided *p*-values of <0.05, were deemed statistically significant. SPSS version 22.0 (SPSS Inc., Chicago, IL, United States) was used to conduct all statistical analyses.

## Results

No significant differences were observed in clinical and preoperative data between the robotic laparoscopy group and the conventional laparoscopy group, as detailed in Table 1. The data revealed that pre-surgical preparation times were longer for patients undergoing robotic laparoscopy compared to those in the conventional laparoscopy group. Conversely, the robotic laparoscopy group exhibited shorter operative durations, more rapid vaginal cuff closures, and reduced estimated blood loss, as shown in Table 2. Furthermore, patients in the robotic group experienced shorter hospital stays, lower Visual Analog Scale (VAS) pain scores, and decreased drainage volumes on the first postoperative day relative to the conventional laparoscopy group, as presented in Table 3.

**Table 1:** Comparison of clinical and preoperative data.

	Laparoscopic (*n*=40)	Robotic (*n*=40)	*p*-value
Age, years	68.7 (4.6)	67.5 (5.3)	0.767
BMI, kg/m^2^	27.2 (7.5)	26.6 (6.7)	0.474
Smokers	7 (17.5)	4 (10.0)	0.518
Previous abdominal surgery	7 (17.5)	6 (15.0)	1.000
FIGO stage			
IA	28 (70.0)	31 (77.5)	0.612
IB	12 (30.0)	9 (22.5)	0.612

Median and standard deviation (SD) and *n* (%) were reported for continuous and categorical variables, respectively. BMI, body mass index; FIGO, the international federation of gynecology and obstetrics.

**Table 2:** Comparison of clinical and intraoperative data.

	Laparoscopic (*n*=40)	Robotic (*n*=40)	*p*-value
Pre-surgical time, min	4.9 (1.3)	10.2 (3.2)	<0.001
Operating time, min	123.4 (34.7)	95.8 (23.4)	<0.001
Vaginal cuff closure time, min	31.3 (9.2)	21.8 (7.5)	<0.001
Estimated blood loss, mL	68.7 (8.8)	50.4 (7.9)	<0.001
Complications	2 (5.0)	1 (2.5)	1.000

Median and standard deviation (SD) and *n* (%) were reported for continuous and categorical variables, respectively.

**Table 3:** Comparison of clinical and postoperative data.

	Laparoscopic (*n*=40)	Robotic (*n*=40)	*p*-value
Hospital stay, days	8.8 (0.9)	7.4 (1.4)	0.008
VAS score	4.8 (2.0)	3.5 (1.2)	0.005
Time to first postoperative flatus, hr	35.2 (11.7)	30.2 (8.2)	0.079
Drainage volume on the first day after operation, ml	244.4 (66.2)	201.2 (48.0)	0.006
Adverse events during hospitalization	5 (12.5)	2 (5.0)	0.432
Adverse events after discharge	6 (15.0)	2 (5.0)	0.263
Infectious adverse events after discharge	5 (12.5)	2 (5.0)	0.432

Median and standard deviation (SD) and *n* (%) were reported for continuous and categorical variables, respectively. VAS, visual analog scale.

**Figure 1:**
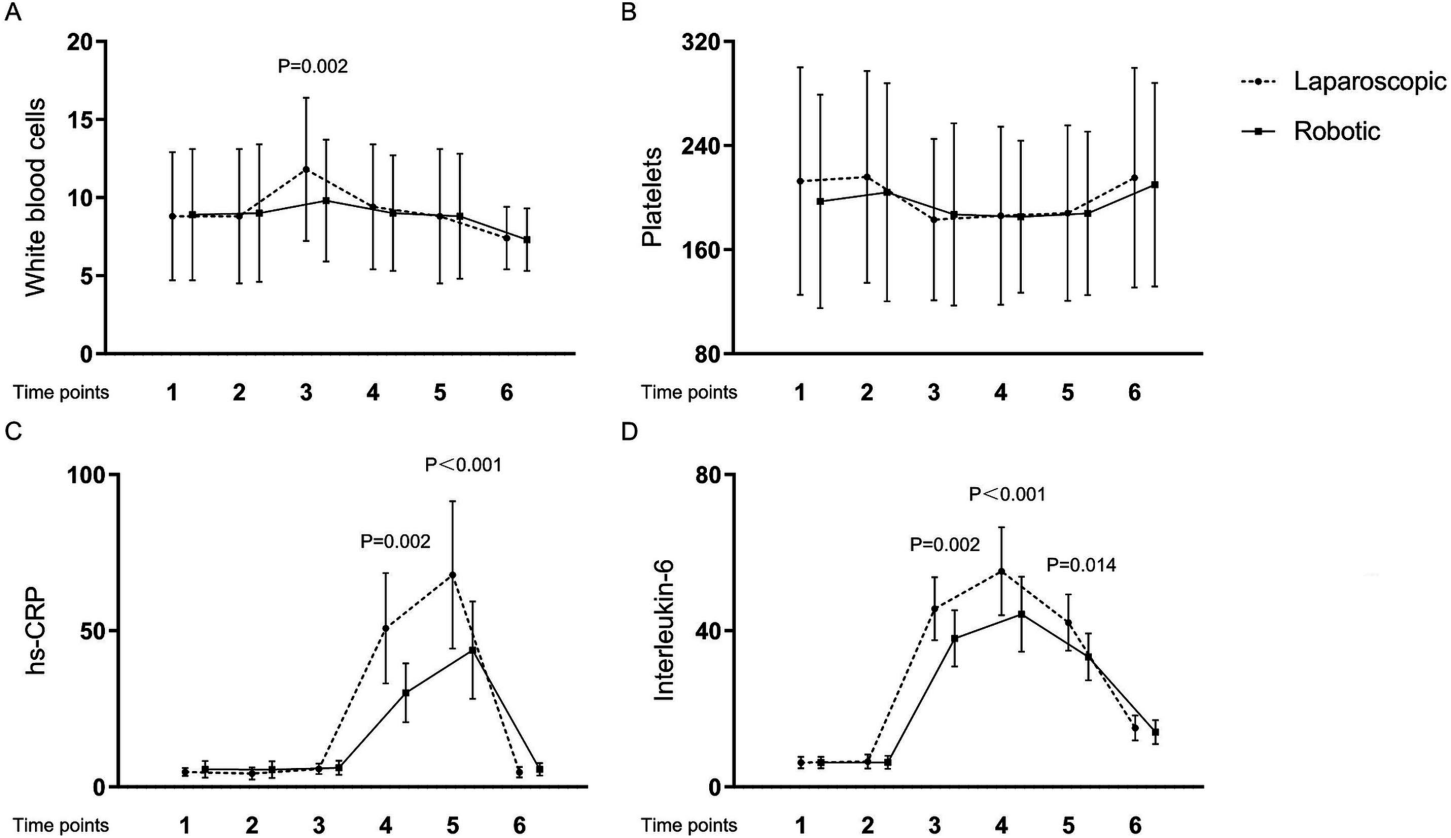
Levels and changes of inflammatory markers over time. (A) white blood cells; (B) platelets; (C) high-sensitivity C-reactive protein; (D) interleukin-6. Blood samples were collected at multiple time points: at admission (Time 1), on the surgery day before the procedure (Time 2), and post-surgery at 2 h (Time 3), 24 h (Time 4), 48 h (Time 5), and 1 week (Time 6).

**Figure 2:**
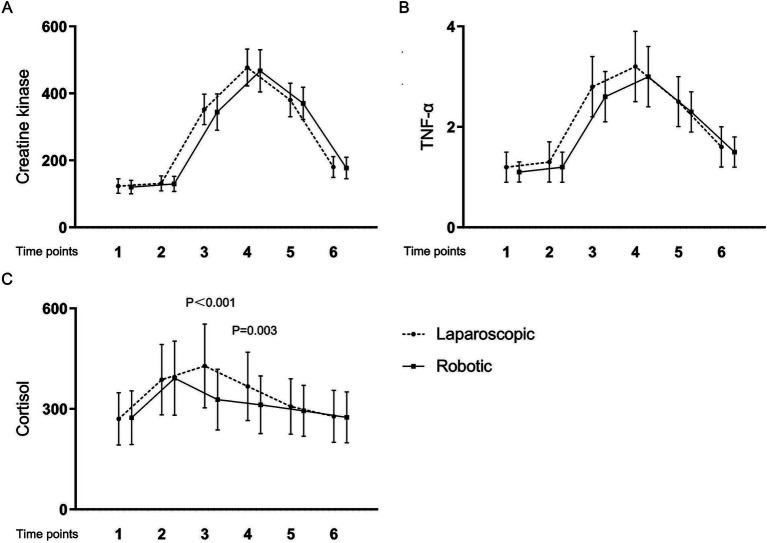
Levels and changes of tissue damage markers over time. (A) creatine kinase; (B) tumor necrosis factor-*α*; (C) cortisol. Blood samples were collected at multiple time points: at admission (Time 1), on the surgery day before the procedure (Time 2), and post-surgery at 2 h (Time 3), 24 h (Time 4), 48 h (Time 5), and 1 week (Time 6).

Table 4 and Figures 1, 2 present the levels and temporal changes of various biomarkers. All markers showed variable trend over time. Regarding inflammatory markers, the conventional laparoscopy group exhibited significantly elevated WBC counts at Time 3 compared to the robotic group. Additionally, IL-6 levels were significantly lower in the robotic group than in the laparoscopic group at Time 3 and Time 4. hs-CRP levels also were significantly reduced in the robotic group compared to the laparoscopic group at Time 4 and Time 5. In terms of biomarkers indicative of tissue damage, cortisol levels were notably lower in the robotic group relative to the laparoscopic group at Time 3 and Time 4.

**Table 4:** Levels and changes of biomarkers.

	Time 1	Time 2	Time 3	Time 4	Time 5	Time 6
WBC, ×10^9^						
Laparoscopic	8.8 (4.1)	8.8 (4.3)	11.8 (4.6)^*^	9.4 (4.0)	8.8 (4.3)	7.4 (2.0)
Robotic	8.9 (4.2)	9.0 (4.4)	9.8 (3.9)	9.0 (3.7)	8.8 (4.0)	7.3 (2.0)
Platelets, ×10^9^						
Laparoscopic	212.7 (87.5)	215.8 (81.6)	183.0 (62.1)	186.0 (68.4)	188.0 (67.5)	215.3 (84.5)
Robotic	197.2 (82.0)	204.2 (83.9)	187.2 (70.0)	185.3 (58.4)	187.8 (62.8)	209.8 (78.2)
hs-CRP						
Laparoscopic	4.8 (1.3)	4.3 (1.9)	5.8 (1.7)	50.8 (17.7)^*^	67.9 (23.6)^*^	4.7 (1.7)
Robotic	5.6 (2.7)	5.5 (2.7)	6.1 (2.3)	30.1 (9.4)	43.8 (15.6)	5.6 (2.0)
Creatine kinase, U/L						
Laparoscopic	123.4 (21.7)	131.4 (22.5)	352.4 (45.6)	477.3 (55.0)	380.0 (50.0)	180.4 (31.3)
Robotic	120.4 (20.2)	129.7 (22.3)	344.0 (54.1)	467.1 (63.2)	370.1 (48.0)	177.1 (32.0)
IL-6, ng/L						
Laparoscopic	6.2 (1.5)	6.5 (1.8)	45.6 (8.1)^*^	55.2 (11.3)^*^	42.1 (7.2)^*^	15.1 (3.2)
Robotic	6.2 (1.5)	6.3 (1.7)	38.0 (7.2)	44.2 (9.6)	33.3 (6.0)	14.0 (3.1)
TNF-α, μg/L						
Laparoscopic	1.2 (0.3)	1.3 (0.4)	2.8 (0.6)	3.2 (0.7)	2.5 (0.5)	1.6 (0.4)
Robotic	1.1 (0.2)	1.2 (0.3)	2.6 (0.5)	3.0 (0.6)	2.3 (0.4)	1.5 (0.3)
Cortisol, mmol/L						
Laparoscopic	270.4 (78.3)	387.2 (104.8)	428.2 (124.9)^*^	367.2 (102.0)^*^	307.2 (82.4)	277.7 (77.4)
Robotic	273.5 (80.1)	391.5 (110.6)	327.7 (90.3)	312.4 (86.3)	294.4 (76.0)	274.7 (75.9)

Median and standard deviation (SD) and *n* (%) were reported for continuous and categorical variables, respectively. WBC, white blood cells; hs-CRP, high-sensitivity C-reactive protein; IL, interleukin; TNF, tumor necrosis factor. ^*^ Significant difference between laparoscopic and robotic groups.

## Discussion

This study demonstrated that robotic laparoscopy for early endometrial cancer yields notably diminished postoperative levels of inflammatory and tissue damage markers, specifically hs-CRP, WBC, IL-6, and cortisol, when compared to conventional laparoscopy. Nonetheless, the disparity in marker levels between the two surgical approaches was transient. This transience was particularly conspicuous for acute inflammatory markers such as WBC, IL-6, and cortisol, which returned to baseline within the initial 24 h post-surgery. Similarly, hs-CRP levels, indicative of a more delayed response, reverted to baseline within 1 to 2 days following the operation.

The participants in this study exhibited relatively elevated body mass index (BMI) levels, measured at 26.9 ± 7.1 kg/m^2^. Extensive research has established a strong correlation between obesity and endometrial cancer, with the risk escalating in tandem with increased BMI (11). The process of estrogen aromatization by adipose tissue, coupled with subsequent stimulation of endometrial proliferation by androgens, represents a primary mechanism driving endometrial carcinogenesis (12). Moreover, the interplay between obesity and heightened estrogen levels can engender a self-perpetuating cycle, further fueling the progression of endometrial cancer (13). Notably, for obese patients, the constrained operative field inherent in conventional laparoscopy poses an exacerbated drawback (14).

Compared to conventional laparoscopy, robotic laparoscopy using the da Vinci system significantly reduces operative time and intraoperative bleeding, benefiting from the following features: (i) the most significant advantages of the da Vinci surgical system lie in its broader range of motion and superior ergonomics. The use of “wristed” instruments enhances precision and flexibility. The robotic arms can rotate 540 degrees, providing a range of motion that not only surpasses that of traditional laparoscopic instruments but also greatly exceeds the capabilities of the human hand (15). (ii) a key distinction between robotic surgery and traditional laparoscopic surgery is that during robotic operations, the surgeon does not directly contact the surgical instruments. In conventional laparoscopic surgery, the instruments are directly manipulated by the surgeon’s hands, thus amplifying any tremors due to hand instability, which is a disadvantage of traditional laparoscopic techniques (16).

In terms of postoperative recovery, this study demonstrates that, compared to the laparoscopy group, the da Vinci robotic group shows significant advantages in terms of time to ambulation, pain scores, drainage volume on the first postoperative day, duration of abdominal drain placement, and length of hospital stay. These benefits underscore the higher safety profile of robotic laparoscopy and indirectly reflect the high precision and dexterity of the robotic “wristed” instruments, thereby reducing surgical trauma (17). These data suggest that for the surgical treatment of endometrial cancer, the da Vinci surgical robot can more effectively reduce both the duration of surgical procedures and the postoperative recovery time for patients, facilitating a quicker postoperative recovery (18).

Our results, revealing heightened inflammatory responses characterized by significantly elevated levels of hs-CRP, WBC, and IL-6, align with those of previous trials (4, 8). These trials prospectively investigated inflammatory responses and other nutritional biomarkers in endometrial cancer patients, illustrating a distinct reaction to surgical trauma induced by open, laparoscopic, or robotic interventions.

WBC counts and hs-CRP are critical biomarkers in assessing the body’s inflammatory response. WBC are a key component of the immune system, rising in response to infections and tissue damage, thus signaling acute inflammatory processes. hs-CRP, synthesized by the liver in response to factors released by macrophages and adipocytes, serves as a sensitive marker of inflammation and has been used to assess the overall severity and progression of inflammatory states (19). Elevations in WBC and hs-CRP levels post-surgery are indicative of the body’s acute phase response to surgical stress and tissue injury. Monitoring these levels can help clinicians assess the severity of inflammation and guide postoperative management to mitigate complications (20). Thus, WBC and hs-CRP are not only markers of the body’s inflammatory state but also reflect the impact of surgical interventions on the body, highlighting their utility in evaluating the body’s stress response to surgical trauma.

IL-6, detectable in plasma as early as one-hour post-tissue injury, stimulates the synthesis and release of C-reactive protein and serves as a primary cytokine in initiating acute inflammatory responses. It is widely regarded as a reliable indicator of surgical trauma severity (21). In this investigation, we observed significant early releases of IL-6, with marked differences manifesting between the robotic and traditional abdominal surgery groups within 2 hours postoperatively. The greater increase in IL-6 levels in the abdominal group suggests more extensive tissue damage. These observations are consistent with prior research comparing inflammatory responses in robotic versus open colorectal surgeries (22, 23).

Cortisol, a key hormone released in response to stress, plays a pivotal role in the body’s physiological response to surgical trauma. Surgical procedures often lead to tissue damage, triggering a cascade of inflammatory and wound healing processes (24). Elevated cortisol levels have been consistently observed in patients undergoing surgery, reflecting the activation of the hypothalamic–pituitary–adrenal (HPA) axis (25). This surge in cortisol levels is believed to modulate various aspects of tissue repair and inflammation, thus potentially serving as a predictive factor for the extent of postoperative tissue damage (26). The mechanisms underlying cortisol’s impact on tissue damage are multifaceted. Cortisol exerts immunomodulatory effects, influencing the balance between pro-inflammatory and anti-inflammatory processes. Additionally, cortisol regulates metabolism, which may influence the availability of nutrients necessary for tissue repair. Moreover, cortisol can impair collagen synthesis and fibroblast function, crucial processes in wound healing, further exacerbating tissue damage (27). In clinical practice, assessing cortisol levels alongside traditional markers of surgical outcome could enhance the prediction of postoperative tissue damage (28). This approach may facilitate early identification of patients at higher risk of complications, allowing for timely interventions to optimize postoperative care and improve patient outcomes. However, further research is warranted to elucidate the specific mechanisms linking cortisol to tissue damage and to validate its utility as a predictive biomarker in surgical settings.

In addition to above considerations, it is crucial to highlight the role of nodal status as one of the most significant prognostic factors in endometrial cancer. Nodal status is well-established as a critical prognostic factor for patients with endometrial cancer, providing essential information to tailor postoperative treatments. However, the role of retroperitoneal staging, which includes pelvic and para-aortic lymphadenectomy, remains controversial. Two independent randomized trials have indicated that pelvic lymphadenectomy does not improve survival rates but increases morbidity in early-stage endometrial cancer patients, prompting a reevaluation of its necessity.

Recent advances have introduced sentinel lymph node (SLN) mapping as a less invasive alternative to comprehensive lymphadenectomy. SLN mapping, associated with lower morbidity, still provides crucial staging information. According to a study (29), SLN mapping does not negatively impact 5-year outcomes in high-intermediate and high-risk endometrial cancer patients, suggesting it can replace lymphadenectomy without compromising prognostic accuracy. Another study (30) emphasizes the need for further prospective trials to validate the long-term benefits and limitations of SLN mapping compared to no nodal staging.

The cumulative evidence supports transitioning from extensive lymphadenectomy to SLN mapping for retroperitoneal staging in endometrial cancer. SLN mapping provides accurate nodal status assessment while minimizing patient morbidity. Future research should focus on refining SLN mapping techniques, improving detection rates, and understanding the molecular and genomic characterization of nodal disease in endometrial cancer.

### Limitation

This study has several limitations that should be acknowledged. Firstly, it was a single-center study with a relatively small sample size, which may limit the generalizability of the findings to broader populations. Secondly, the time points chosen for the collection of blood samples for inflammatory and tissue damage markers were based on convenience for the clinical research protocol, rather than comprehensive coverage of the peak levels of each biomarker. This means that the true metabolic patterns of these indicators may not have been fully captured. Additionally, some biomarkers may exhibit delayed responses, which could have been missed given the predetermined sampling schedule. These limitations should be considered when interpreting the results of this study.

## Conclusion

This study illustrated the changes in markers of inflammation and tissue damage following robot-assisted laparoscopy and traditional laparoscopy. It further established that robotic hysterectomy, when used in early endometrial cancer treatment, results in a reduced inflammatory response, diminished tissue damage, and a lesser stress reaction, as indicated by lower levels of hs-CRP, IL-6, and cortisol, compared to abdominal hysterectomy in similar cases. While the variations in these markers were transient, the reduced tissue damage observed in the robotic group may lead to a quicker improvement in patient-reported health-related quality of life, a finding we have previously reported.

## Data Availability

The raw data supporting the conclusions of this article will be made available by the authors, without undue reservation.
